# The Contribution of Real-Time Artificial Intelligence Segmentation in Maxillofacial Trauma Emergencies

**DOI:** 10.3390/diagnostics15080984

**Published:** 2025-04-12

**Authors:** Amjad Shhadeh, Shadi Daoud, Idan Redenski, Daniel Oren, Adeeb Zoabi, Fares Kablan, Samer Srouji

**Affiliations:** 1 Department of Oral and Maxillofacial Surgery, Galilee College of Dental Sciences, Galilee Medical Center, Nahariya 2210001, Israel; 2 The Azrieli Faculty of Medicine, Bar-Ilan University, Safed 1311502, Israel

**Keywords:** artificial intelligence, AI in clinical decision support, auto-segmentation, maxillofacial trauma, emergency medicine, real-time imaging, AI-based diagnostic imaging, AI-supported treatment planning

## Abstract

**Background/Objectives:** Maxillofacial trauma poses significant challenges in emergency medicine, requiring rapid interventions to minimize morbidity and mortality. Traditional segmentation methods are time-consuming and error-prone, particularly in high-pressure settings. Real-time artificial intelligence (AI) segmentation offers a transformative solution to streamline workflows and enhance clinical decision-making. This study evaluated the potential of real-time AI segmentation to improve diagnostic efficiency and support decision-making in maxillofacial trauma emergencies. **Methods:** This study evaluated 53 trauma patients with moderate to severe maxillofacial injuries treated over 16 months at Galilee Medical Center. AI-assisted segmentation using Materialise Mimics Viewer and Romexis Smart Tool was compared to semi-automated methods in terms of time and accuracy. The clinical impact of AI on diagnosis and treatment planning was also assessed. **Results:** AI segmentation was significantly faster than semi-automated methods (9.87 vs. 63.38 min) with comparable accuracy (DSC: 0.92–0.93 for AI; 0.95 for semi-automated). AI tools provided rapid 3D visualization of key structures, enabling faster decisions for airway management, fracture assessment, and foreign body localization. Specific trauma cases illustrate the potential of real-time AI segmentation to enhance the efficiency of diagnosis, treatment planning, and overall management of maxillofacial emergencies. The highest clinical benefit was observed in complex cases, such as orbital injuries or combined mandible and midface fractures. **Conclusions:** Real-time AI segmentation has the potential to enhance efficiency and clinical utility in managing maxillofacial trauma by providing precise, actionable data in time-sensitive scenarios. However, the expertise of oral and maxillofacial surgeons remains critical, with AI serving as a complementary tool to aid, rather than replace, clinical decision-making.

## 1. Introduction

Emergency departments are complex systems characterized by high patient volume, unpredictable patient arrivals, and a wide range of medical conditions. The complexity of this environment can lead to challenges in providing efficient and effective care [1,2]. Maxillofacial trauma is a prevalent and significant concern in emergency medicine, posing challenges for both patients and healthcare providers. These issues can lead to substantial morbidity, disability, and even mortality if not properly managed. In many cases, immediate intervention is essential for effective treatment [3,4,5].

The evolution of trauma imaging began with plain radiographs, offering basic two-dimensional views of fractures. This advanced with computed tomography (CT) and cone beam computed tomography (CBCT), providing sectional imaging and detailed views of internal structures across multiple planes. Three-dimensional CT reconstruction further improved the spatial orientation of anatomical structures [6]. More recently, CT segmentation has improved diagnostic accuracy by isolating specific regions of interest, facilitating the analysis of complex cases. Features like transparency and selective removal of certain structures improve our understanding of intricate anatomy, leading to more precise and informed clinical decisions [7,8].

Artificial Intelligence (AI) has made significant advancements in healthcare, including in emergency medicine, where it shows great potential in improving diagnosis and treatment efficiency within time-sensitive decision-making environments [9]. Diagnostic AI algorithms have accelerated diagnostic and therapeutic processes, often matching or surpassing clinical experts’ performance [10]. Traditionally, manual segmentation has been time-consuming and dependent on practitioner expertise. Semi-automated segmentation, while faster, relies on threshold selection and often requires manual adjustments, making it both time-intensive and prone to human error [11,12,13]. Additionally, within conventional segmentation methods, the presence of metal artifacts significantly increases the workload, potentially leading to practitioner fatigue, with any errors in accuracy negatively impacting surgical outcomes [14]. In contrast, automated segmentation has demonstrated improved performance, rapidly identifying anatomical structures, fractures, and other regions of interest, providing a more time-efficient and consistent alternative under clinician supervision and review [15,16].

Artificial intelligence-based segmentation has been increasingly applied in maxillofacial surgery, particularly for diagnostic and preoperative planning purposes [16]. Morita et al. [14] employed a 2D U-Net architecture for facial bone segmentation, demonstrating technical feasibility. Yeshua et al. [17] developed a deep learning model for 3D segmentation of bone lesions in CBCT images, and Oliveira-Santos et al. [18] used AI for automated mandibular canal segmentation to support implant planning workflows. In the context of reconstructive surgery, Yan et al. [19] applied AI to design patient-specific guide plates, streamlining complex aspects of symmetry and shape optimization. Collectively, these studies demonstrate the expanding role of AI in elective and image-based applications in maxillofacial surgery.

Recent studies have explored the application of AI in trauma-related maxillofacial diagnostics, particularly for fracture detection. Multiple convolutional neural network (CNN) models have been developed to identify mandibular fractures in panoramic radiographs, achieving high diagnostic accuracy and, in some cases, surpassing expert-level human performance [20,21]. Tong et al. [22] reported comparable results using a CNN-based approach for the detection of zygomatic fractures on CT imaging. In addition, AI has been introduced as a reliable tool for the CT-based diagnosis of orbital blowout and nasal bone fractures [23,24,25], further demonstrating its potential to enhance diagnostic accuracy and efficiency in facial trauma care. While these studies highlight the promising diagnostic capabilities of AI, they primarily address detection tasks and do not evaluate the clinical application of AI-assisted segmentation in real-time emergency settings. In contrast, the present study investigates the integration of AI segmentation tools within the acute care workflow, focusing on both performance and perceived clinical utility in the management of complex maxillofacial trauma.

This study aims to highlight the efficiency and the potential contributions of real-time AI segmentation in improving the diagnosis and management of maxillofacial emergencies based on our department’s experience.

The remainder of this paper is organized as follows: Section 2 describes the segmentation workflow, study design, and evaluation methods. Section 3 presents the experimental results and analysis. Section 4 discusses the findings in relation to the existing literature and highlights limitations. Finally, Section 5 concludes the study by summarizing the main findings and their implications for clinical practice.

## 2. Materials and Methods

### 2.1. Study Design and Imaging Workflow

This prospective study was conducted over a 16-month period in the Department of Oral and Maxillofacial Surgery at Galilee Medical Center, Nahariya, Israel. The study included 53 adult trauma patients (>18 years) who presented to the emergency department with moderate to severe maxillofacial injuries involving displaced fractures of the facial bones that necessitated surgical intervention for reduction and fixation. All patients had undergone CT imaging as part of the standard trauma assessment protocol. Exclusion criteria included cases involving cranial vault fractures, simple non-displaced fractures deemed insufficiently complex for segmentation evaluation, and patients with a known history of prior facial trauma.

The types of fractures observed in the study population were categorized as follows: isolated mandible fractures (19 cases), isolated midface fractures (16 cases), combined mandible and midface fractures (11 cases), and pure orbital fractures (7 cases).

Trauma management followed Advanced Trauma Life Support (ATLS) protocols, with initial stabilization focusing on securing the airway, supporting breathing, controlling active bleeding, and maintaining hemodynamic stability. CT scans were performed using the SOMATOM X.cite CT scanner (Siemens Healthineers, Erlangen, Germany) with a slice thickness of 1 mm. The generated DICOM images were imported into AI-enabled segmentation tools.

The study was approved by the Institutional Review Board of Galilee Medical Center and conducted in accordance with the principles of the Declaration of Helsinki.

### 2.2. Segmentation Procedure

The segmentation process focused on identifying key anatomical structures essential for trauma management, including the airway, mandible, midface, teeth, and foreign bodies. AI-assisted segmentation was conducted using one of two tools: Materialise Mimics Viewer (version 2.2.43.12) or the Smart Tool of Romexis (version 6.4), both of which are FDA-approved for clinical medical use. For comparative analysis, semi-automated segmentation was performed using Materialise Mimics Core 26.0, utilizing threshold-based segmentation followed by manual refinement, including region growing and slice-by-slice editing of anatomical structures if needed, carried out by an oral and maxillofacial surgery resident trained in advanced segmentation workflows. The time required for each segmentation method was recorded, starting from the upload of the CT DICOM file into the software to the point at which the segmentation results were displayed on the screen.

**Figure 1:**
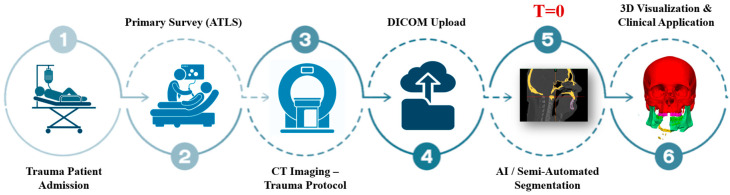
Clinical workflow of AI-assisted segmentation in maxillofacial trauma. Trauma patients undergo initial evaluation following Advanced Trauma Life Support (ATLS) guidelines, followed by CT imaging under the trauma protocol. The resulting DICOM files are uploaded into segmentation platforms, where AI and/or semi-automated tools generate anatomical models. These are then used for rapid 3D visualization to support diagnosis, airway evaluation, and surgical planning. T = 0 marks the point of successful DICOM upload, from which segmentation time is calculated.

The complete workflow from trauma admission to AI-assisted planning is summarized in Figure 1, where segmentation time was measured beginning at the point of successful DICOM upload (T = 0).

### 2.3. Assessment

Segmentation accuracy was assessed by comparing AI-generated outputs and semi-automated segmentations to ground truth segmentations, which were created and validated by two experts in oral and maxillofacial imaging. Accuracy was quantified using three complementary metrics:

1. Dice Similarity Coefficient (*DSC*):

This metric measures the overlap between the segmented region and the ground truth. It is defined as

$$DSC=\frac{2*TP}{2*TP+FP+FN}$$

where *TP* (True Positives) are regions correctly identified as part of the ground truth. *FP* (False Positives) are regions incorrectly identified as part of the segmentation but not in the ground truth. *FN* (False Negatives) are regions in the ground truth that were missed by the segmentation.

*DSC* provides a reliable measure of segmentation performance, with higher values indicating greater accuracy and alignment with the ground truth; a value of 1 represents a perfect match.

2. Jaccard Index (JI):

This metric quantifies the intersection-over-union between predicted and ground truth segmentations, offering additional sensitivity to partial overlaps. It is defined as

$$\mathrm{JI}=\frac{\left|A\cap B\right|}{\left|A\cup B\right|}$$

where *A* and *B* represent the sets of voxels from the predicted and ground truth segmentations, respectively.

3. Hausdorff Distance (*HD*):

HD measures the maximum boundary deviation between the segmented surfaces. It is defined as

$$HD\left(A,B\right)=\max\left\{\underset{a\in A}{\sup}\underset{b\in B}{\inf\;}|a-b|,\;\underset{b\in B}{\sup}\underset{a\in A}{\inf\;}|b-a|\right\}$$

where $\left\|a-b\right\|$ denotes the Euclidean distance between surface points aaa and bbb. Lower HD values indicate better geometric alignment.

While DSC was calculated across the full dataset, JI and HD were evaluated on a representative subset of 28 trauma cases to provide additional insight into spatial and geometric segmentation performance. All metrics were computed using dedicated 3D analysis software (Materialise 3-matic Medical (version 19.0) and 3D Slicer (version 5.8.1)).

### 2.4. Contribution Evaluation

The AI-powered segmentation results for the various cases were evaluated by two maxillofacial experts to determine their clinical utility in the diagnostic and treatment processes, with each expert assessing the segmentation using a 5-point Likert scale, ranging from 1, indicating no clinical utility, through 2, reflecting minimal utility with negligible clinical impact, 3, representing moderate utility with clear clinical relevance, 4, denoting high utility that significantly aids in clinical decision-making, and 5, signifying essential utility, indispensable for clinical management. For each case, the evaluations from the two experts were averaged to calculate a mean score. Additionally, to quantify the overall utility of auto-segmentation across all fracture types, the scores were summed and averaged. The clinical contribution to both diagnosis and treatment planning was assessed based on these evaluations.

### 2.5. Statistical Analysis

Unpaired *t*-tests were applied to compare the segmentation times between the AI-assisted and semi-automated methods. The results were considered statistically significant at a *p*-value < 0.05. Descriptive statistics were used to summarize the accuracy metrics, with results presented as means and standard deviations (SD).

## 3. Results

**Figure 2:**
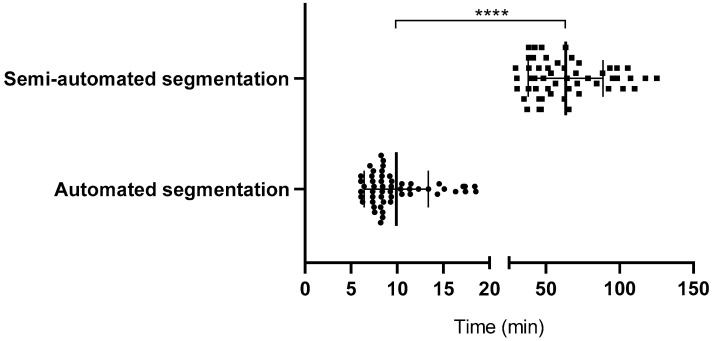
Time comparison between automated and semi-automated segmentation. Bars indicate mean ± SD. Circles represent individual cases segmented using automated (AI-assisted) methods, while squares represent cases segmented using semi-automated methods. Significance was determined by a two-tailed unpaired *t*-test (**** *p* < 0.0001).

This study included 53 trauma patients presenting with moderate to severe maxillofacial injuries in an emergency setting, with a mean age of 37.47 ± 12.92 years, comprising 38 men and 15 women. The segmentation performance of AI-assisted tools was compared to that of semi-automated methods, emphasizing time-efficiency and accuracy. The AI-assisted segmentation method demonstrated a significant improvement in time-efficiency (*p* < 0.0001), with an average time of 9.87 ± 3.45 min, compared to 63.38 ± 25.07 min for the semi-automated approach, making it approximately 6 times faster (Figure 2)**.** The effect size for this comparison, calculated using Cohen’s d, was 2.99, indicating a large and clinically meaningful difference in segmentation time.

Quantitative analysis of 28 representative trauma cases confirmed strong agreement between AI-generated and expert-defined segmentations. Materialise Mimics Viewer achieved a Jaccard Index of 0.89 ± 0.03 and a Hausdorff Distance of 0.91 ± 0.13 mm, while Planmeca Romexis Smart Tool yielded a Jaccard Index of 0.88 ± 0.04 and a Hausdorff Distance of 0.93 ± 0.15 mm. Semi-automated segmentation performed slightly better in spatial metrics, with a Jaccard Index of 0.91 ± 0.03 and a Hausdorff Distance of 0.88 ± 0.11 mm.

The Dice Similarity Coefficient (DSC), calculated across the full dataset, was 0.93 ± 0.03 for auto-segmentation using Materialise Mimics Viewer, 0.92 ± 0.02 for Planmeca Romexis Smart Tool, and 0.95 ± 0.02 for the semi-automated method, all reflecting high overlap with ground truth segmentations. Despite the semi-automated method achieving slightly higher numerical scores, the difference in overall accuracy between the two approaches was not statistically significant. AI-based segmentation remained within clinically acceptable limits and offered a significant reduction in processing time.

**Figure 3:**
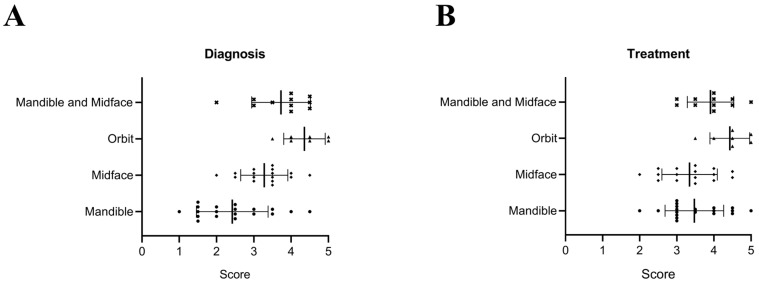
Contribution of AI-assisted segmentation to diagnosis (**A**) and treatment planning (**B**) across different types of fractures. The scores represent clinical utility, rated on a scale from 1 (no utility) to 5 (essential utility). Bars indicate mean ± SD. ● indicates mandibular fractures, ◆ indicates midface fractures, ▲ indicates orbital fractures, and × indicates combined mandibular and midface fractures.

In addition, the clinical utility of AI-assisted segmentation in diagnostic and treatment workflows was assessed by two oral and maxillofacial experts using a 5-point Likert scale (Figure 3). For orbital fractures, AI segmentation received a score of 4.4/5 for both diagnosis and treatment planning, highlighting its substantial contribution in these cases. Similarly, for combined mandible and midface fractures, the utility was rated at 3.7/5 for diagnosis and 3.9/5 for treatment planning, demonstrating its value in managing complex anatomical structures and enhancing clinical workflows. Across all fracture types, the mean utility scores were 3.18/5 for diagnosis and 3.65/5 for treatment planning and decision-making.

The AI-powered segmentation produced high-quality 3D visualizations of critical anatomical regions, providing real-time data that facilitated rapid decision-making in the management of facial trauma. These 3D visualizations effectively highlighted key anatomical structures such as the airway, mandible, midface, teeth, and foreign bodies.

The following sections will present specific examples of various trauma cases included in this study. These examples illustrate the potential of real-time AI segmentation to enhance the efficiency of diagnosis, treatment planning, and overall management of maxillofacial emergencies.

### 3.1. Rapid Airway Assessment

**Figure 4:**
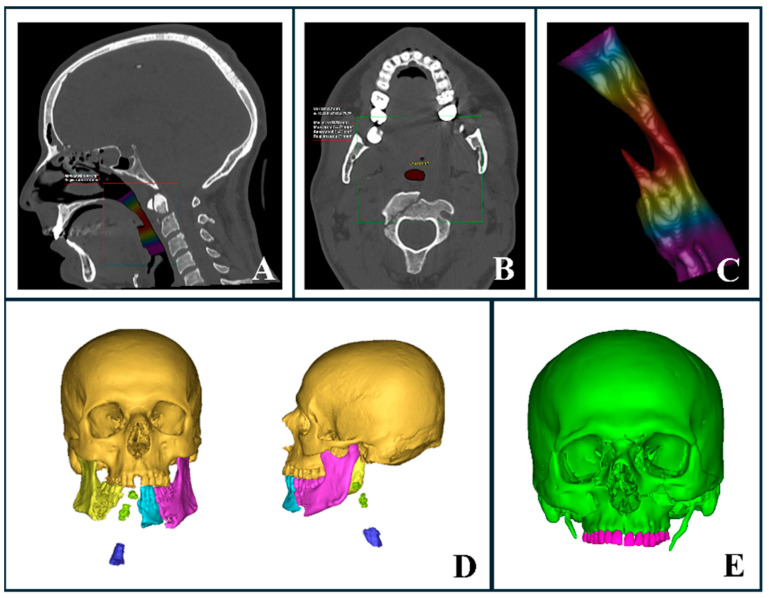
AI-segmented upper airway and trauma evaluation. (**A**–**C**) Automated upper airway segmentation and analysis in a trauma patient with a complex mandibular fracture. (**A**) Sagittal CT section highlighting airway volume. (**B**) Axial CT section displaying the minimal inner airway area. (**C**) Three-dimensional representation of the airway with a heat map indicating varying thicknesses, where red marks the narrowest regions. (**D**) Three-dimensional reconstruction illustrating airway compromise due to severe mandibular trauma and loss of bony support. (**E**) Three-dimensional automated segmentation showing a complex midface fracture with clear involvement of the naso-orbito-ethmoid (NOE) region.

Airway compromise in facial trauma can result from factors such as edema, bleeding, loss of bony support, displacement of bone fragments, and the presence of foreign bodies. AI-powered segmentation enabled the rapid assessment of these factors, providing critical information for emergency upper airway management, including volumetric analysis and minimal inner surface area evaluation (Figure 4A–C). While CT imaging is typically performed only after primary stabilization, which may include airway management when necessary, AI segmentation proved particularly useful in assessing airway patency in trauma patients. Among the patients evaluated, 12 required intubation to secure the airway prior to undergoing CT imaging, underscoring the importance of rapid decision-making in these critical cases.

In cases where intubation was not initially required, AI-generated 3D segmentation offered clinicians precise and reliable evaluations of the airway, facilitating the early detection of factors that could compromise the airway, such as foreign bodies. This allowed clinicians to intervene promptly and secure the airway if required. In this study, none of the trauma patients initially evaluated without intubation required subsequent airway intervention following AI segmentation and airway analysis. It is important to note that airway volume assessment and minimal area measurements can vary significantly, depending on factors such as patient positioning, age, anatomy, and background [26,27,28]. Since there are no established studies providing definitive upper airway averages for trauma patients, these measurements were used for supplementary assessment rather than as definitive determinants.

Additionally, detailed visualizations of injury severity and location helped clinicians determine the most appropriate intubation method prior to surgery under general anesthesia, whether performed immediately or within a few hours of admission to the emergency department. For instance, a severely comminuted mandibular fracture compromising the airway may necessitate a tracheostomy (Figure 4D). Although nasal intubation is commonly utilized in oral and maxillofacial surgery, it may be contraindicated in specific trauma scenarios. In cases involving naso-orbito-ethmoid (NOE) or cranial base fractures, nasal intubation should be avoided in favor of oral or submental intubation (Figure 4E).

### 3.2. Complex Facial Fractures

CT imaging provided a detailed three-dimensional assessment of complex maxillofacial fractures, allowing for the precise evaluation of fracture type, location, severity, and fragment displacement. AI-driven segmentation significantly accelerated the visualization of these injuries, aiding our surgical planning and decision-making processes in the fast-paced emergency department.

**Figure 5:**
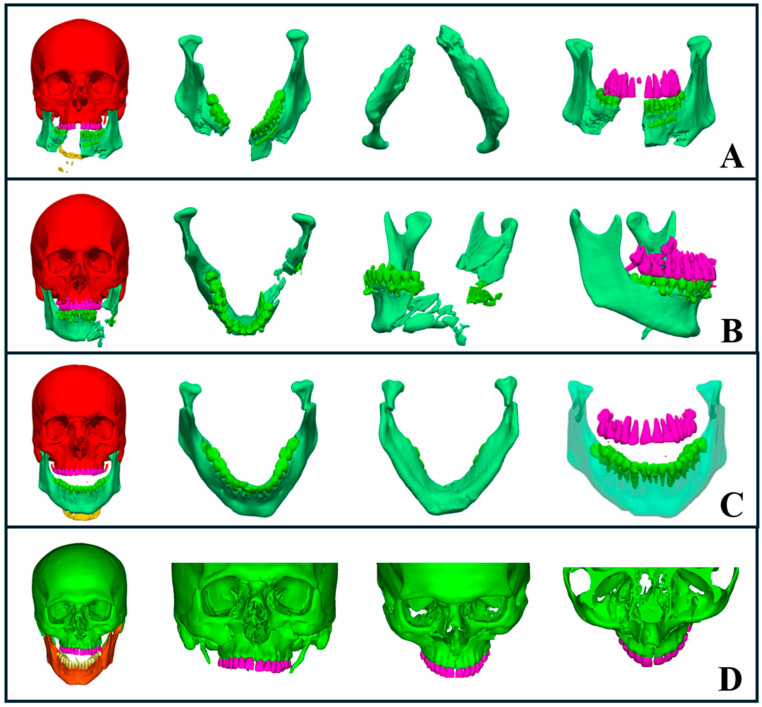
AI-driven segmentation of complex maxillofacial fractures. (**A**) Severe avulsion and comminuted mandibular fractures with evident mandibular widening. (**B**) Comminuted fracture of the left mandibular body and ramus, including occlusion evaluation. (**C**) Bilateral subcondylar fractures with medial displacement, highlighting an open bite. (**D**) Comminuted midface fracture involving the zygomaticomaxillary complex, midpalatal region, naso-orbito-ethmoid area, and orbital floor, with segmentation showing fracture severity and extent.

Mandibular fractures pose particular challenges due to the mandible’s U-shaped structure and the biomechanical forces involved, complicating treatment. Open fractures often require rapid immobilization to minimize infection risk and ensure adequate perfusion. Using AI-powered segmentation, we were able to perform a swift and detailed assessment of fracture patterns, which guided early interventions and helped anticipate potential complications. For severe avulsion fractures with mandibular widening (Figure 5A), AI segmentation expedited our decisions regarding the appropriate airway management technique and the optimal reconstruction approach. In cases of comminuted mandibular fractures (Figure 5B), AI-segmented regions of interest assisted us in determining the most suitable reduction and fixation methods. For condylar fractures (Figure 5C), the enhanced 3D segmentation helped us visualize the displacement patterns and decide between open or closed treatment approaches.

Midface fractures, including naso-orbito-ethmoid, zygomatic complex, maxillary, and orbital fractures, pose additional challenges due to their anatomical complexity and proximity to vital structures. AI-driven segmentation allowed us to rapidly identify fracture patterns, dislocations, and avulsions, leading to accurate classification and treatment decisions. Additionally, it assisted in planning airway management when needed, particularly in cases where intubation required careful consideration (Figure 5D).

Orbital fractures are critical as they affect both visual function and esthetics, potentially requiring immediate intervention. AI segmentation of orbital bones from CT scans enabled a rapid and precise assessment of fracture severity, which helped us determine the need for surgical intervention. For example, fractures involving more than 50% of the orbital floor with ocular symptoms, such as diplopia, necessitated surgical repair (Figure 5D). AI segmentation provided clear visualizations that guided our choice of surgical approach, identified intact orbital walls, and helped determine the appropriate plate type and fixation methods.

### 3.3. Teeth Auto-Segmentation

Artificial intelligence techniques, particularly deep learning models, have shown great potential in automating the segmentation of teeth from CT scans [29,30,31,32,33,34]. In the emergency setting, teeth segmentation was crucial for rapidly assessing dental injuries and planning surgical treatments. Accurate segmentation helped us evaluate occlusion, which influenced our decisions between closed treatment with intermaxillary fixation and open reduction with internal fixation (Figure 5A–C).

AI segmentation allowed us to quickly assess the available space apical to tooth roots, which assisted in determining the optimal placement for plates or screws. Additionally, it helped evaluate tooth bone support, particularly when considering the use of teeth as anchors for teeth-borne arches. In cases where teeth were located within the fracture line, the segmentation and 3D visualization enabled us to make more precise treatment decisions, improving both planning and surgical outcomes.

### 3.4. Foreign Body Auto-Segmentation

**Figure 6:**
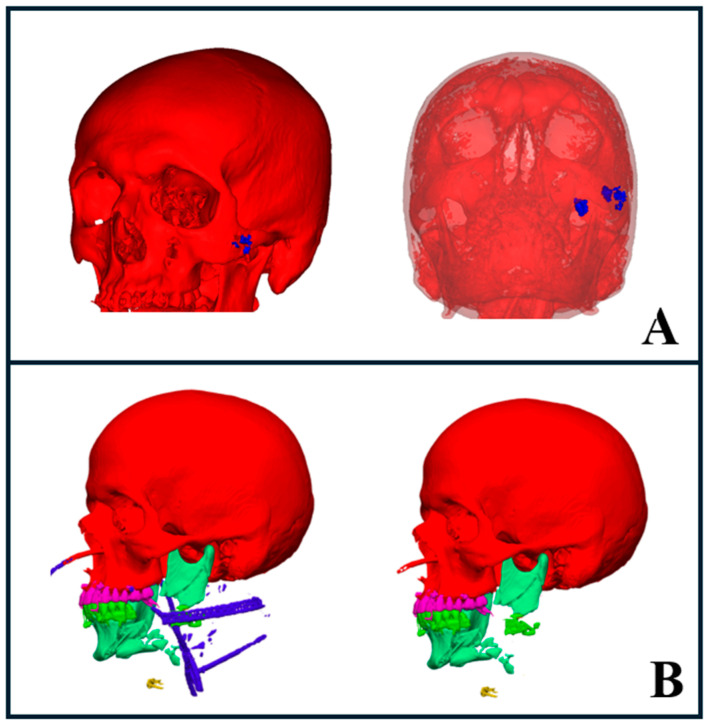
AI segmentation of foreign bodies and trauma hardware. (**A**) Gunshot wound case showing segmented foreign bodies (blue) on the left side of the face (left image), with transparency applied to bony structures revealing deeper foreign bodies in the infratemporal region (right image). (**B**) Severe trauma case with segmented hardware, including intubation lines and drains, highlighted in blue (left image). The right image shows the same case with hardware elements hidden, providing a clearer view of the underlying anatomy.

AI-driven metal segmentation is crucial in managing maxillofacial injuries, particularly in cases like gunshot wounds, as it allows for the precise identification and localization of foreign bodies, aiding in treatment decisions [8]. In our study, segmentation enabled us to accurately pinpoint foreign bodies, which helped determine the need for immediate removal when they compromised vital anatomical structures such as the airway, nerves, or other essential areas (Figure 6A).

We also used AI segmentation to assess the positioning of metal objects, drains, and surgical hardware, which helped us determine the extent of surgery required for their removal. This facilitated the design of the surgical approach while enabling us to anticipate potential complications. Additionally, the software provided the ability to temporarily hide these segmented objects in 3D visualizations. This feature gave us a clearer view of the underlying fractures, which may improve the treatment planning and decision-making processes (Figure 6B).

### 3.5. Stereolithography Model Fabrication

**Figure 7:**
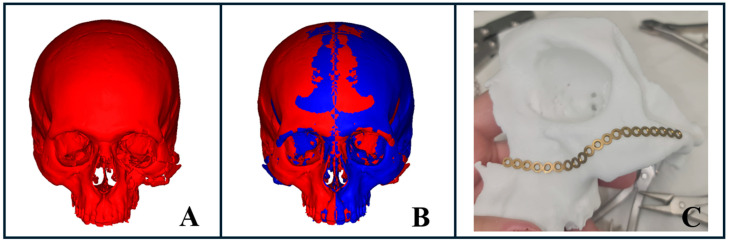
Stereolithographic model fabrication for complex facial fractures. (**A**) AI-powered segmentation illustrating a severe midface fracture with spatial deformity of the zygomaticomaxillary complex. (**B**) Virtual surgical planning using mirroring of the healthy side to project the desired fracture reduction, followed by file export for 3D printing. (**C**) Pre-bent plate prepared using the 3D-printed model based on the mirrored healthy side, demonstrating final preparation for surgical intervention.

AI-driven segmentation significantly accelerated the creation of precise 3D models for complex facial fractures (Figure 7A), which proved especially valuable in emergency settings. These models supported us in both preoperative planning and intraoperative procedures by providing enhanced visualization of detailed anatomy and improving overall treatment efficiency.

For trauma cases requiring rapid intervention, this technology optimized the workflow, from segmentation to virtual surgical planning, including virtual reduction or mirroring of the unaffected side, to model fabrication (Figure 7B,C). This process enabled us to pre-bend plates within just a few hours (Figure 7C), facilitating more accurate reconstruction and efficient treatment outcomes.

## 4. Discussion

The integration of real-time AI-driven segmentation in maxillofacial emergency care may offer clear advantages, particularly in enhancing diagnostic speed and accuracy [16]. Our findings suggest that by automating the segmentation of key anatomical structures such as the mandible, midface, teeth, and airway, AI can support clinicians in making faster, more informed decisions regarding primary surgical interventions, which is critical in time-sensitive emergencies.

Additionally, AI-driven segmentation reduced the time clinicians spend on manual or semi-manual segmentation, allowing them to focus on higher-value tasks. It also provides consistent and reliable results, minimizing the variability and human errors often associated with manual or semi-manual methods, particularly in the fast-paced environment of emergency departments. Moreover, with AI-enabled tools like Materialise Mimics Viewer, the segmented data are accessible via an online platform. This allows colleagues to review the trauma segmentation in detail, add comments at specific locations, and collaborate on treatment planning.

The outcomes of this study suggest that real-time AI segmentation can be effectively integrated into clinical workflows, particularly in trauma centers where CT imaging is routinely employed for emergency care. The platforms evaluated, Materialise Mimics Viewer and Romexis Smart Tool, are commercially available and currently in clinical use at our institution. Both AI segmentation tools and the software used for semi-automated segmentation were installed on the same high-performance workstation (Intel Core i7, 32 GB RAM) and operated within a secure hospital network. Segmentation time was recorded beginning from successful DICOM upload. While AI-assisted segmentation was significantly faster overall, a slight variability in processing time was observed in cases affected by metallic artifacts, suboptimal image quality, or complex anatomical disruption. Although this workflow is feasible in well-equipped clinical environments, broader adoption in resource-limited settings may be challenged by factors such as software licensing, infrastructure requirements, and staff training needs.

Integrating real-time AI segmentation into emergency workflows presents both practical and ethical challenges. Ensuring data privacy, particularly the anonymization of CT data, is essential for regulatory compliance. Technically, the process depends on high-performance workstations, secure data infrastructure, and stable connectivity. Successful implementation also requires trained clinical staff proficient in segmentation tools and the interpretation of AI outputs. For centers utilizing 3D printing, access to appropriate hardware and technical support is needed.

Although AI-assisted segmentation yielded slightly lower accuracy values across all metrics, including the DSC, JI, and HD, these differences were not statistically significant and did not affect clinical decision-making in our cohort. Nonetheless, certain scenarios, such as complex comminuted fractures, overlapping anatomical structures, or the presence of metal artifacts, can present challenges to AI performance. In such cases, manual refinement or expert validation may still be required to ensure accurate segmentation and safe clinical application. In light of these considerations, future studies involving larger and more diverse patient cohorts will be important to further validate and generalize these findings.

A number of studies have explored software-assisted 3D modeling in maxillofacial surgery, primarily in the context of elective procedures such as anatomical segmentation for surgical planning, implant design, or lesion evaluation, using semi-automated or AI-based techniques [14,16,17,18,19]. Other recent studies have applied AI to trauma diagnostics, particularly for the detection of mandibular, zygomatic, nasal, and orbital fractures using panoramic radiographs and CT imaging, with several models achieving diagnostic performance comparable to expert interpretation [20,21,22,23,24,25]. While these works underscore the growing role of AI in both surgical planning and trauma diagnostics, they are largely focused on retrospective detection tasks or static image analysis. In contrast, the present study investigates the real-time clinical integration of AI-assisted segmentation within emergency workflows, emphasizing its time efficiency and practical utility in the acute management of complex facial trauma.

Despite AI’s promising capabilities, several challenges remain [9]. Different AI algorithms and software platforms may produce inconsistent results, especially if not trained on a diverse set of data. The lack of standardization across AI systems means that the accuracy of segmentation can vary depending on the tool being used, potentially leading to treatment delays or misdiagnosis. Additionally, integrating AI into existing healthcare systems presents logistical and financial hurdles, such as the need for proper resource allocation and the development of user-friendly interfaces. Despite automation, AI segmentation requires careful review by clinicians. Errors in AI output, such as misidentifying anatomical structures or fractures, could lead to poor treatment decisions if unchecked. Therefore, AI should be seen as a complementary tool, enhancing but not replacing the expertise of oral and maxillofacial surgeons.

## 5. Conclusions

This study demonstrates the clinical feasibility and value of integrating real-time AI-assisted segmentation into emergency workflows for maxillofacial trauma management. Quantitatively, AI tools significantly reduced segmentation time (mean 9.87 ± 3.45 min vs. 63.38 ± 25.07 min for semi-automated tools, *p* < 0.0001) while maintaining high accuracy across multiple metrics, including DSC (0.92–0.93), JI (0.88–0.89), and HD (0.91–0.93 mm). Qualitatively, expert evaluations confirmed the clinical utility of AI-generated models, with high diagnostic usefulness scores, particularly for orbital trauma and complex treatment planning. These findings support the integration of AI segmentation tools as a time-saving and reliable adjunct to existing clinical workflows in high-pressure trauma settings. Although implementation requires appropriate infrastructure and trained personnel, the results affirm that AI can enhance diagnostic speed and consistency without compromising accuracy. As AI technologies continue to mature, their role in supporting decision-making and optimizing outcomes in maxillofacial surgery is likely to expand.

## Data Availability

The data used to support this study’s findings are available by contacting the corresponding author upon reasonable request.
